# Vascular toxicities with VEGF inhibitor therapies–focus on hypertension and arterial thrombotic events

**DOI:** 10.1016/j.jash.2018.03.008

**Published:** 2018-06

**Authors:** Rhian M. Touyz, Sandra M.S. Herrmann, Joerg Herrmann

**Affiliations:** a Institute of Cardiovascular and Medical Sciences, BHF Glasgow Cardiovascular Research Centre, University of Glasgow, Glasgow, Scotland, United Kingdom; b Division of Nephrology and Hypertension, Department of Medicine, Mayo Clinic, Rochester, MN, USA; c Department of Cardiovascular Diseases, Mayo Clinic, Rochester, MN, USA

**Keywords:** Angiogenesis inhibitors, cardiovascular events, chemotherapy, hypertension

## Abstract

The vascular endothelial growth factor (VEGF) signaling pathway (VSP) fulfills a cardinal role in endothelial cells and its inhibition has profound cardiovascular impact. This is true not only for the normal vasculature but also for the tumor vasculature when VSP inhibitors are used as anti-angiogenic therapies. Generalized endothelial dysfunction predisposes to vasoconstriction, atherosclerosis, platelet activation, and thrombosis (arterial more than venous). All of these have been reported with VSP inhibitors and collectively give rise to vascular toxicities, the most concerning of which are arterial thromboembolic events (ATE). VSP inhibitors include antibodies, acting extracelluarly on VEGF, such as bevacizumab and tyrosine kinases inhibitors, acting intracellularly on the kinase domain of VEGF receptors, such as sunintib and sorafenib. The addition of bevacizumab and VSP tyrosine kinase inhibitor therapy to the cancer treatment regimen is associated with a 1.5–2.5-fold and 2.3–4.6-fold increase risk of ATEs, respectively. Risk factors for ATEs while on VSP inhibitor therapy include age older than 65 years, previous thromboembolic events, history of atherosclerotic disease, and duration of VSP inhibitor therapy. In clinical practice, hypertension remains the most commonly noted vascular manifestation of VSP inhibition. Optimal blood pressure goals and preferred therapeutic strategies toward reaching these goals are not defined at present. This review summarizes current data on this topic and proposes a more intensive management approach to patients undergoing VSP inhibitor therapy including Systolic Blood PRessure Intervention Trial (SPRINT) blood pressure goals, pleiotropic vasoprotective agents such as angiotensin converting enzyme inhibitors, amlodipine, and carvedilol, high-dose statin therapy, and aspirin.

## Highlights

• Increase in blood pressure is nearly universal with VEGF inhibitor therapy.
• Optimal blood pressure goals and preferred therapeutic strategies are not defined.
• Systolic Blood Pressure Intervention Trial target goals are justifiable in VEGF inhibitor-induced hypertension, given the high arterial vascular event risk.
• Patients on VEGF inhibitor therapy may benefit from cardio-/nephro-oncology care.

## Graphical abstract

Central to the vascular effects of vascular endothelial growth factor (VEGF) inhibitors are the actions on endothelial cell level. Anti-VEGF therapy targets not only endothelial cell proliferation and permeability, which is the goal to antagonize the formation of new tumor vessels, but also endothelial cellsurvival, vasorelaxation, and platelet activation. The end results are systemic hypertension, accelerated atherosclerosis, and arterial thrombotic events. Various interventions are geared to promote endothelial and vascular health and to reduce the detrimental effects of VEGF inhibitor therapy while maintaining its anti-angiogenic efficacy.

**Figure:**
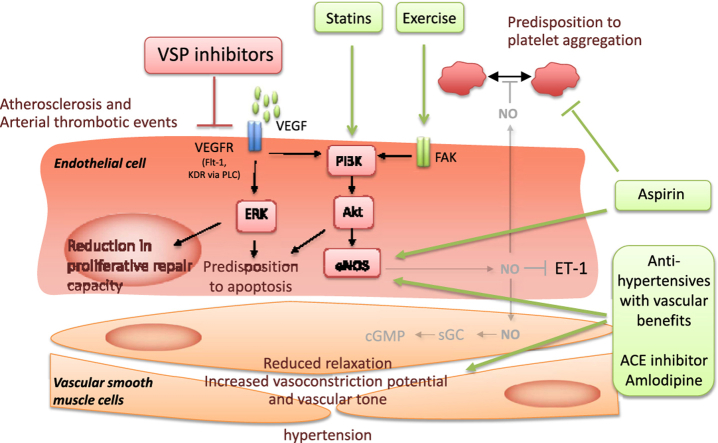

The concept that growth of tumors is related to their vascular supply was first described over 100 years ago.^1^, ^2^ In 1971, Judah Folkman^1^, ^2^ suggested that tumorigenesis and metastasis are dependent on the formation of new blood vessels (angiogenesis) and that blocking angiogenesis could be a strategy to inhibit tumor progression. Vascular endothelial growth factor (VEGF) is one of the most important growth factors to promote angiogenesis and changes in the tumor microenvironment. In agreement, drugs that block the VEGF signaling pathway (VSP) have expanded the therapeutic options for several solid tumor cancers, such as metastatic colorectal cancer, non-small cell lung cancer, and gliobastoma.^3^, ^4^, ^5^ One of the most classic examples, however, is metastatic renal cell carcinoma (mRCC), for which VSP inhibitors have doubled response and overall survival rates.^6^ By 2016, the US Food and Drug Administration approved seven antiangiogenic drugs as either first- or second-line therapy for mRCC, all of these targeting the VEGF signaling. VSP inhibitors, including sorafenib (Nexavar, Bayer), sunitinib (Sutent, Pfizer), bevacizumab (Avastin, Genentech), pazopanib (Votrient, Novartis), axitinib (Inlyta, Pfizer), cabozantinib (Cometriq, Exelixis), and lenvatinib (Lenvima, Eisai) have been especially effective in renal cell carcinoma (RCC) based on the link with the von Hippel-Lindau gene/protein. This tumor suppressor gene/protein targets hypoxia-inducible factor (HIF), the transcription factor involved in VEGF expression, to ubiquitin-mediated proteasomal degradation and its inactivation (which leads to increased HIF and thus VEGF levels) has been implicated in the pathoetiology of (clear) RCC.^7^

Since angiogenesis does not initiate but is involved in the maintenance and dissemination of the malignant process, angiogenesis inhibitors, for the most part, contain, but do not eliminate, cancers. This unique efficacy goes along with long, chronic treatment times, and evolving toxicities can become quite relevant for the individual cancer patient.^8^ Growing evidence demonstrates that cancer patients treated with VSP inhibitors, including direct VEGF inhibitors such as anti-VEGF antibodies or decoy receptors, and small molecule VEGF tyrosine kinase inhibitors (TKIs), are at increased risk of developing cardiovascular disease (CVD). Adverse vascular and cardiac events are rarely a reason for discontinuation of VSP inhibitor therapy, but they are important causes for fatal outcomes. In fact, 25%–66% of all fatal events in VSP-treated cancer patients are vascular in nature, especially hypertension, arterial thromboembolism, myocardial infarction, and cerebrovascular disease.^9^ Worth to mention in this context is that semaxinib, the first oral VSP-TKI to enter clinical trials, was withdrawn after a high rate of thromboembolism was noted in combination therapies (42% arterial thromboembolic events [ATEs] when combined with gemcitabine and cisplatin and 25% VTEs when combined with paclitaxel).^10^, ^11^, ^12^

A recent meta-analysis of 77 studies reported that over 1200 patients need to be treated with VSP inhibitors for one fatal event to occur, and overall survival is not reduced.^13^ Such data might argue against the greater clinical relevance of these cardiovascular (CV) side effects in a cancer population in need of therapy. At the very least, these data make the point that any untoward side effects must be balanced against the perceived benefit. As such, familiarity with these events is important as is the appropriate management. Figure 1 illustrates the risks of CV events in terms of numbers needed to harm,^13^ and this review will focus on the vascular toxicities of VSP inhibitor therapy, related to hypertension, altered vascular reactivity, accelerated atherosclerosis, and acute arterial events. A summary of incidences of vascular events with VSP inhibitors and chemotherapeutics in general is provided in Table 1.

**Figure 1:**
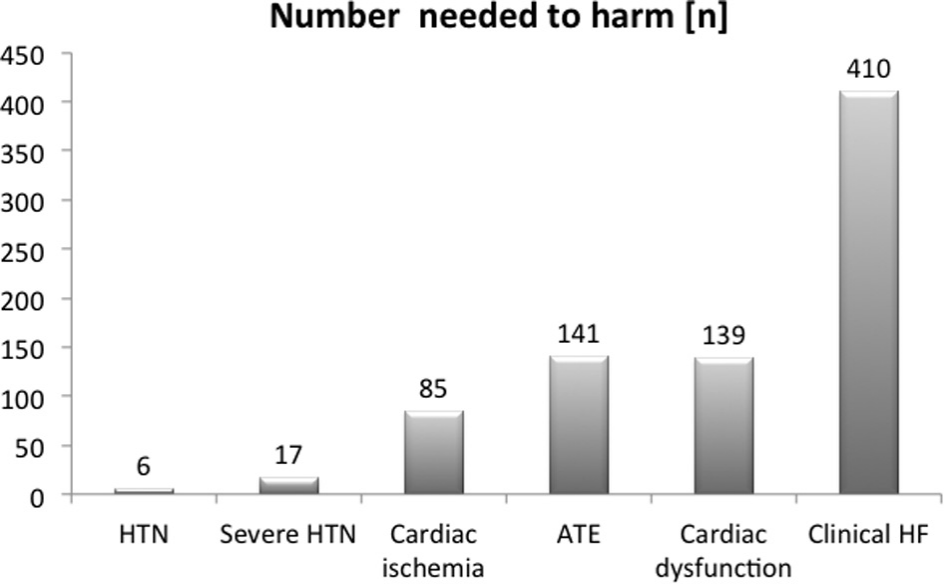
Bar graph illustrating the number needed to treat to cause one of the indicated CV events (based on data from^13^). HTN, hypertension; ATE, arterial thromboembolic events; HF, heart failure.

**Table 1:** Incidence of vascular toxicities with chemotherapeutics (based on Micromedex and Lexicomp)

Type of cancer therapy	HTN (%)	Angina (%)	AMI (%)	Raynaud's (%)	Stroke (%)	PAD (%)	Pulm HTN (%)	DVT/PE (%)
Antimetabolites								
5-Fluorouracil		ND	ND	ND	ND			
Capecitabine	<1	≤6	1–10	<1	<1			<1
Gemcitabine		<1	<1	<1				
Antimicrotubule agents								
Paclitaxel	1	<1	<1					1
Alkylating agents								
Cisplatin		3	2	<1	<1	<1		
Cyclophosphamide		<1	<1				<1	
Antitumor antibiotics								
Bleomycin		<1	<1	<1	<1		<1	
Vinca alkaloids								
Vincristine	ND	ND	ND	ND				
mTOR inhibitors								
Everolimus	4–13	5	<1					1–10
Temsirolimus	7	16						2
Proteasome inhibitors								
Bortezomib	6		ND		ND		ND	ND
Carfilzomib	15–42	3–21	<20				≤2	
Monoclonal antibodies								
Bevacizumab	19–42	8	6 (ATE)		6 (ATE)			6–14
Ramucirumab	11–36		2 (ATE)		2 (ATE)			
Rituximab	6–12	<1	<1					
VEGF-receptor fusion molecules								
Aflibercept	41		3 (ATE)		3 (ATE)			5–9
Tyrosine kinase inhibitors								
Sorafenib	9–41	<1	≤3		<1			<1
Sunitinib	15–34	13	<1		≤1			≤3
Pazopanib	40–42	5–10	≤2		1			5
Axitinib	40	<1	≤2		≤1			1–3
Regorafenib	30–59	≤1	≤1					
Cabozantinib	33–61		≤2 (ATE)		≤2 (ATE)			4–7
Vandetanib	33				1%			
Lenvatinib	42–73		7–10					3–5
Nilotinib	10	5–9	≤1		1–3	3–4		ND
Ponatinib	53–74	21	11–42 (ATE)		11–42 (ATE)	12		2–6
Dasatinib		4					ND	<1
Miscellaneous								
Interferon-alpha 2B	≤9	≤28	<5	<5	<5	<5	<5	<5
Thalidomide								≤23
Lenalidomide	6–8	5–8	≤5		≤2			2–10

AMI, acute myocardial infarction; ATE, arterial thromboembolic events; DVT, deep vein thrombosis; HTN, hypertension; mTOR, mammalian target of rapamycin; ND, frequency not defined; PAD, peripheral arterial disease; PE, pulmonary embolism; VEGF, vascular endothelial growth factor.

## Incidence and Risk Factors for Systemic Hypertension with VSP Inhibitors

Almost all clinical trials have demonstrated that VSP inhibitors cause an increase in blood pressure, with 30%–80% of patients developing hypertension.^14^, ^15^ Initial studies with bevacizumab found a 20%–30% higher than expected risk of hypertension, but the need for (intensification of) antihypertensive therapy was only seen in 10%–20% of patients, and life-threatening hypertensive crisis occurred in only approximately 1% of patients.^16^, ^17^ It is important to recognize that event rates are critically dependent on the definition, assessment, and adjudication of events. For instance, the incidence of hypertension with bevacizumab (all grades) is more than double with home than with office blood pressure measurements (55% vs. 24%).^18^ Home blood pressure monitoring, conducted appropriately after adequate instructions, is more sensitive to detect milder and earlier stages of hypertension. The argument that the use of clinical cancer trial criteria could have confounded the incidences is addressed by the observation that the hypertension criteria of the European Society of Hypertension and the National Cancer Institute Common Toxicity Criteria (NCI-CTC), version 3.0, yielded fairly comparable detection rates in the office-based setting.^18^ It is important though that an appropriately calibrated device with a correctly sized cuff is used, and ideally a minimum of two measurements several minutes apart are taken and averaged.

**Figure 2:**
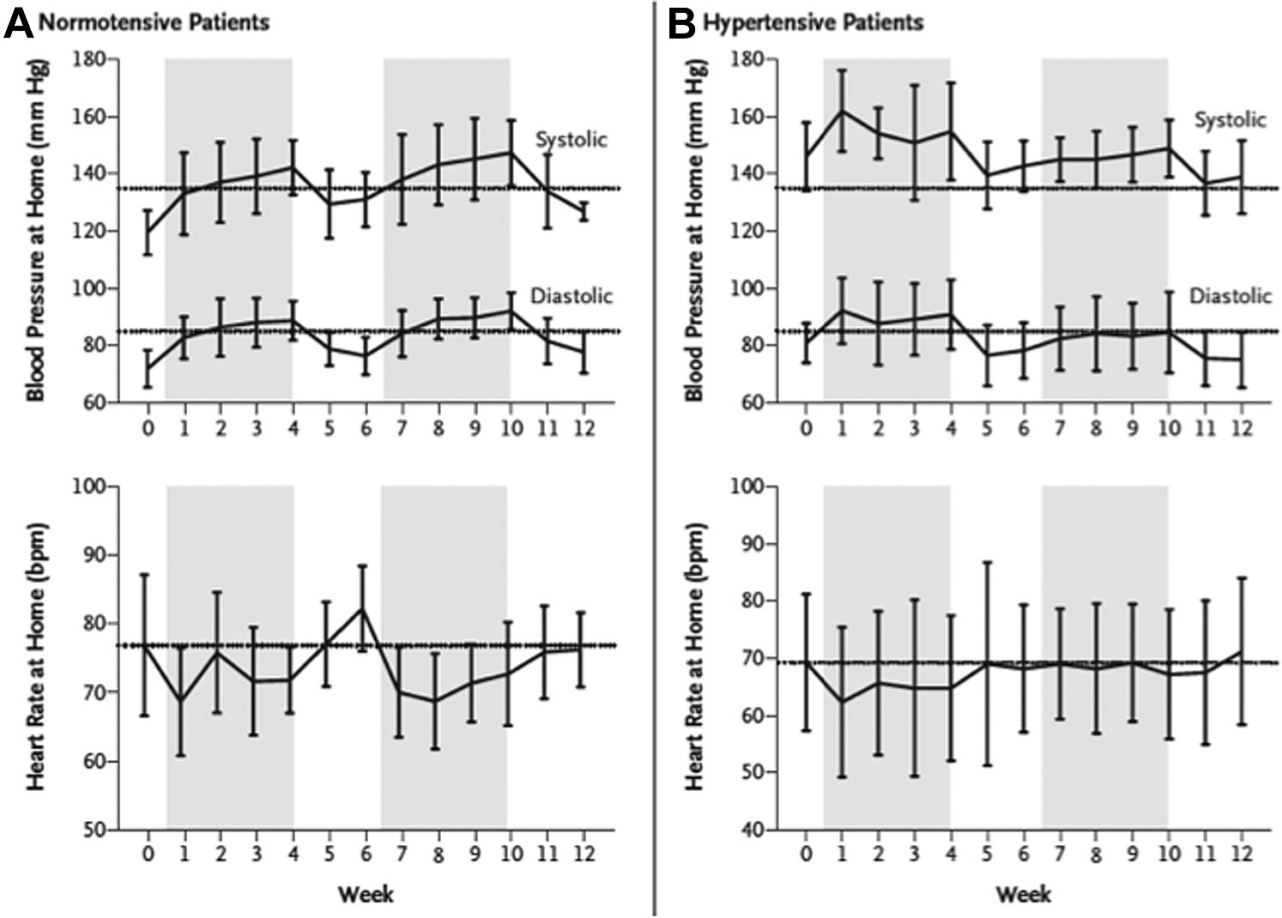
Illustration of systolic and diastolic blood pressures of patients undergoing treatment with sunitinib, stratified by baseline history of hypertension, absent (panel A) or present (panel B).

The first report on automated ambulatory blood pressure monitoring (ABPM) in patients with mRCC on sunitinib showed an average increase in systolic and diastolic blood pressure by 14 mmHg and 11 mmHg, respectively, in the first week and by 22 mmHg and 17 mmHg, respectively, after 4 weeks (Figure 2).^19^ It also demonstrated that following discontinuation of therapy, the blood pressure falls quickly, within 1 week. In initially normotensive patients, the blood pressure may not return to pre-therapy levels after the first cycle; rather it is reset to a new baseline that is several points higher. It is for this reason that higher blood pressure values are reached with the second cycle. Multiple resets are not necessarily seen though, and the dynamics are responsive to antihypertensive therapy. Indeed, adjustment in antihypertensive therapy can fully blunt hypertensive responses on VSP inhibitor therapy.

As delineated in subsequent ABPM studies, increases in blood pressures occur rapidly, often within hours, and circadian blood pressure dynamics are typically lost or significantly attenuated. In a mixed cohort of patients receiving sorafinib, the average increase in blood pressure, within the first day of treatment, was 7/6 mmHg. A further increase was noted during days 6–10, followed by a plateau as steady-state concentrations of the drug equilibrated.^20^ Overall, 93% of patients had an increase in the mean arterial blood pressure (MAP) from 2 to 27 mmHg in the plateau stage. Of these, 28% had an increase less than twice the limit of detection for change, and 16% had an increase two times the average change of 16.7 mmHg of MAP.^20^ Likewise in ovarian cancer patients receiving cedarinib, almost all (95%) of patients had an increase in blood pressure within 3 days but of variable degree (average increase 19 mmHg systolic, 17 mmHg diastolic, MAP increase from 2 to 55 mmHg, most <35 mmHg, in two-thirds of relevant change).^21^

Even within the same study population, exposed to the same treatment regimen, considerable variability in the blood pressure response can be seen. Age ≥60years, body mass index ≥25 kg/m^2^, and pre-hypertension were identified as predictors of a hypertensive response, each adding an absolute 10% increase in risk to a baseline risk without any risk factors of 30%.^22^ Other studies, however, were unable to verify these or any other predictors.^20^ Accordingly, all patients should undergo blood pressure monitoring, especially as this is an easy and inexpensive test.

## Clinical Implications of Systemic Hypertension with VSP Inhibitors

Awareness and monitoring are geared to recognize and appropriately manage blood pressure elevations in cancer patients on VEGF inhibitor therapy. In the general population, blood pressure management serves the purpose of primary (and secondary) prevention of CVD and events long-term. This used to rarely apply to cancer patients with limited exposure and life expectancy. This argument, however, may not hold any more with improvement in survival outcomes and chronicity of cancer treatment.

While often not considered detrimental, even short-term increases in blood pressure can have serious implications in cancer patients on VEGF inhibitor therapy. The consequences include acute ischemic and hemorrhagic events, flash pulmonary edema, and reversible posterior leukoencephalopathy. Chronic blood pressure elevations stimulate a hypertrophic response in the myocardium, which is matched by a compensatory angiogenic response. In cancer patients on VSP inhibitor therapy, this compensatory response is inhibited, predisposing to myocardial maladaption to pressure load and “cardiotoxicity”. Hypertension is a potent risk factor for atherosclerosis, its complications, as well as atrial fibrillation, all of which can complicate the care of cancer patients significantly. Uncontrolled hypertension is conceivably of much greater risk in patients with additional CV risk factors and especially CVD. Interestingly, hypertension with VEGF inhibitor therapy is not a risk factor for proteinuria in patients exposed to this therapy.^21^ This is in agreement with experimental studies pointing out that proteinuria and renal injury including glomerular ischemia develop as a function of VEGF inhibitor dose. Hypertension occurs before proteinuria manifests and is evident at lower doses of VSP inhibitors than those that cause proteinuria.^21^ Hence, hypertension, proteinuria, and renal dysfunction are not causally linked in VSP inhibitor-treated patients.^23^ In RCC patients with solitary kidneys after nephrectomy, these aspects are especially relevant. Renal dysfunction secondary to VSP inhibitor use is beyond the scope of this review but is covered elsewhere.^17^, ^24^

It is also beyond the scope of this review to fully discuss the merit of systemic hypertension as a “biomarker” for the anticancer activity and efficacy of VEGF therapy. For instance, in patients on treatment with sunitinib for mRCC, overall survival was almost 2 years longer in those with systolic blood pressure increases to ≥140 mmHg.^25^ Several other but importantly not all studies have made similar observations. As such, hypertension may serve as a reflection of the on-target effects of VEGF inhibitors in cancer patients and the vascular changes occurring on tumor level.

Another aspect of interest in this regard is the production of VEGF-A splice variants in cancers such as VEGF-A165b.^26^, ^27^ This splice variant is capable of binding to VEGF receptor-2 (R2) but with inhibitory effects, thus decreasing the angiogenic tumor response.^28^ Moreover, VEGF-A165b binds bevacizumab with the same affinity as VEGF-A165, therefore reducing the therapeutic efficacy of bevaciumab as well.^29^ In the presence of circulating VEGF-Axxxb splice variants, the neutralizing effects on bevacizumab would be on a much larger scale. These patients would be expected to have a much less than expected blood pressure increase on therapy. It is not expected that VSP-TKIs would be neutralized by VEGF-Axxxb splice variants in a manner similar to bevacizumab. However, in the presence of VEGF-A165b and its inhibitory effects on VEGF-R2 signaling with reduced activity (phosphorylation) of the receptor tyrosine kinase and activation of downstream signaling pathways, VEGFR-TKIs may not find much of a target. In this setting then again, an attenuation of the treatment effect and possibly vascular effects could be anticipated. Accordingly, one could make an argument that testing for splice variants and quantification of the ratio of VEGF-Axxx to VEGF-Axxxb splice variants may serve as a predictor of vascular side effects with therapy. It would need to be combined with a preparation of what to do therapeutically for these cancer patients. In summary, monitoring blood pressure dynamics in cancer patients on VEGF inhibitor therapy can provide important clues to the molecular tumor environment and response to therapy as it relates to this key angiogenesis factor.

## Mechanisms of Systemic Hypertension with VSP Iinhibitors

**Figure 3:**
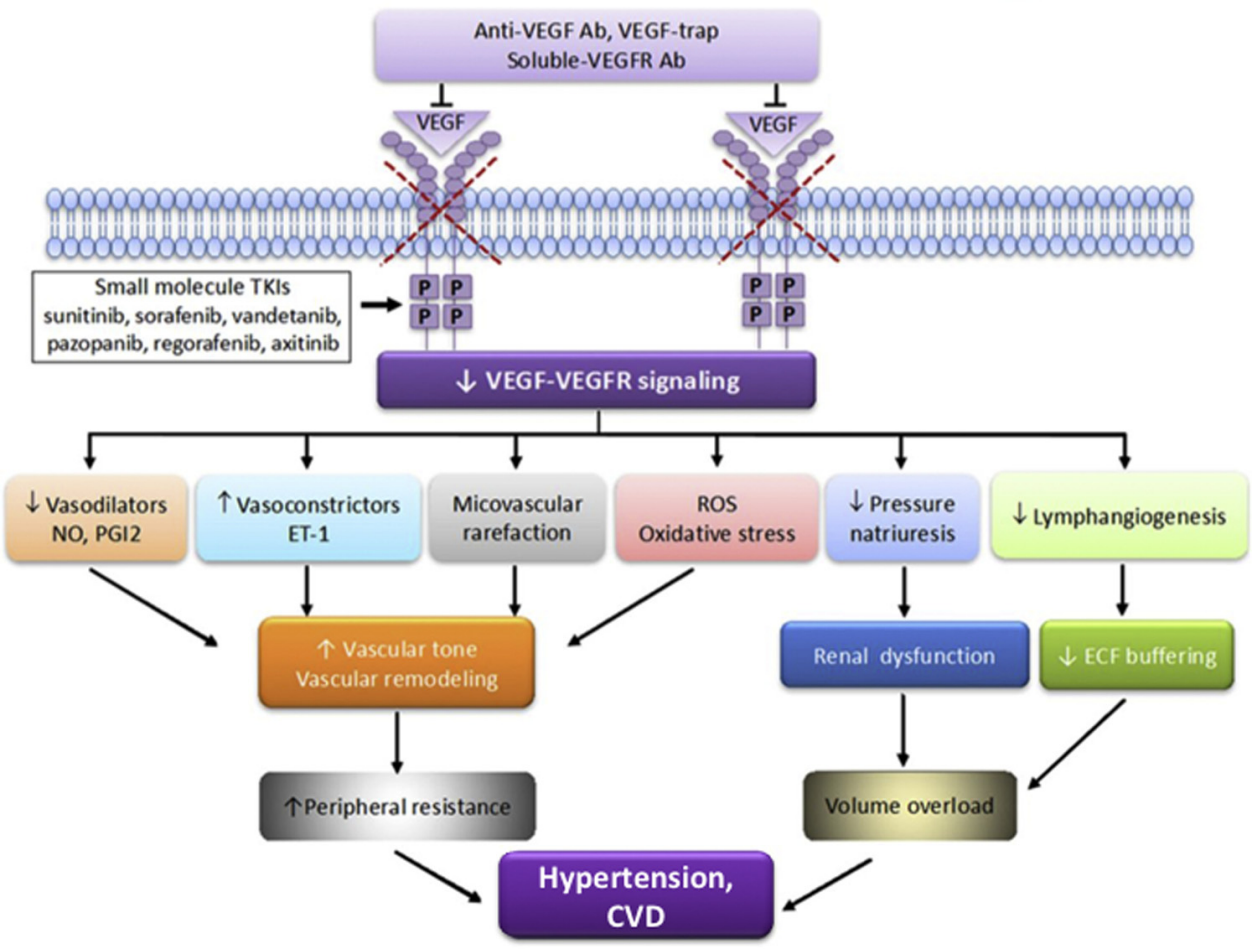
Illustration of possible pathophysiological processes, whereby VEGF (vascular endothelial growth factor)–VEGFR (VEGF receptor) inhibition contributes to the development of hypertension and CVD. Four major classes of VEGF–VEGFR inhibitors, including monoclonal VEGF antibodies, anti–VEGF-R2 antibodies, soluble decoy receptors (VEGF-traps), and small molecule VEGFR tyrosine kinase inhibitors (TKIs), are used clinically as antiangiogenesis drugs in cancer. The effects are reduced VEGFR signaling and consequent reduction in production of vasodilators (NO and PGI2), increased production of vasoconstrictors (ET-1), oxidative stress and rarefaction, resulting in increased vascular tone and arterial remodeling. Reduced pressure natriuresis and impaired lymphatic function contribute to volume overload. Ab, antibody; CVD, cardiovascular disease; ECF, extracellular fluid; ET-1, endothelin-1; NO, nitric oxide; p, phosphorylation site of tyrosine kinase; PGI2, prostaglandin I2; ROS, reactive oxygen species.

Various mechanisms for the development of hypertension with VEGF inhibitor therapy have been suggested (Figure 3). In a classical contribution, Horowitz et al. demonstrated that VEGF administration to live animals results in a predictable hypotensive response, which can be antagonized by inhibition of nitric oxide (NO) synthase (NOS).^30^ Conversely, administration of a VEGF-R2 antibody causes a rapid and sustained increase in blood pressure, and differences between treated and untreated animals are abolished by NOS inhibition.^31^ No activation of the renin-angiotensin system was noted in this setting; rather, endothelial and neuronal NOS expression in the kidney was suppressed. The important role of NO in the regulation of (medullary) renal blood flow and (tubular) sodium excretion has been recognized for a long time and it has been postulated that inhibition or deficiency of NOS would result in sustained hypertension.^30^ Thus, while renal dysfyunction is not the initial cause of hypertension in patients on VEGF inhibitor therapy, inhibition of renal NO signaling is associated with a rightward shift of the renal pressure-natriuresis curve, impaired sodium excretion, and consequently fluid retention and salt-dependent hypertension.^14^

(Modified from Touyz RM, Lang NN, Herrmann J, van den Meiracker AH, Danser AHJ. Recent Advances in Hypertension and Cardiovascular toxicities with VEGF Inhibition. *Hypertension*. 2017; 70:220-226).

While it is clear that VEGF inhibition interferes with NO signaling pathway activity, even in humans, it has remained a matter of debate how the interplay between VSP inhibitors and NO affects the vasculature and its contributions to blood pressure elevations.^32^ In vascular terms, the cardinal sign of reduced NO production and/or availability is endothelial dysfunction. This condition is classically recognized by impairment of endothelium-dependent vasodilation in response to acetylcholine. In healthy volunteers, that is, in the absence of any comorbid condition and treatment that could alter the response, bevacizumab at clinically relevant treatment concentrations very specifically and acutely (within 15 minutes) reduced endothelium-dependent vasodilation.^33^ No increase in vascular tone or blood pressure was noted in this particular study, and subsequent work showed that impairment of endothelium-dependent vasodilation does not precede the development of hypertension in patients on sunitinib arguing against a direct causal link.^34^ In agreement, while tivozanib and telatinib reduced flow-mediated dilatation in patients over a treatment period of 8 and 6 weeks, respectively, vandetanib over 6 weeks had no such effect even though it did decrease plasma nitrate/nitrite levels and resting brachial artery diameter and all drugs significantly increased systemic blood pressures.^35^, ^36^, ^37^ Clearly, the impact of VSP-TKIs is much more complex and likely related to the additional “off-target” effects. Along these lines, bevacizumab is a much “cleaner” drug, and reports confirm similar impairment of flow-mediated dilation after 6 weeks of bevacizumab therapy as well as increase in pulse wave velocity indicative of increased arterial stiffness.^38^ As such, there is a definite effect of VEGF inhibition on the vasculature, and as outlined in other experimental studies, the net effect that can be linked to blood pressure elevation is an increase in systemic vascular resistance.^39^ Stimulation of the endogenous endothelin (ET) system seems to relate to these changes even if the endothelium is not the source of the elevated ET-1 levels, which have been confirmed in patients as well.^40^ One may argue that capillary rarefaction, observed in animal models and patients on VSP inhibitor therapy, is a responsible factor for the increased systemic resistance. However, it has been estimated that a 40% reduction of fourth order vessels is required for a 5% increase in vascular resistance, and it seems very unlikely for this to occur over the course of a few hours.^39^ As seen in patients with idiopathic (essential) hypertension, capillary rarefaction may be the consequence rather than the cause of hypertension in patients undergoing VEGF inhibitor therapy.^33^ This is not to say though that eventually it contributes to the sustained blood pressure elevations. Likewise, it cannot be ruled out that a declining NO activity is unmasking the activity of the endogenous ET system, and that oxidative stress has a permissive or contributory role.^33^, ^41^, ^42^ As a final point, much of what has been discussed mirrors discussions and findings of women with preeclampsia. A common link would be the high levels of soluble VEGF-R1 in these women, which sequesters VEGF in a manner similar to bevacizumab and afilbercept.^15^

## Monitoring and Management of Systemic VSP Inhibitor-Induced Hypertension

There is currently no formal guideline on the type of blood pressure monitoring in patients on VSP inhibitor therapy (office single reading vs. office average of multiple readings vs. home monitoring vs. ABPM). The Cardiovascular Toxicities Panel, Convened by the Angiogenesis Task Force of the National Cancer Institute Investigational Drug Steering Committee, recommended that blood pressures should be monitored weekly during the first cycle of VEGF inhibitor therapy, and once stable blood pressures are achieved, depending on the level of risk for complications, the evaluation schedule might be more conveniently aligned with home blood pressure monitoring or routine clinical evaluations, at least every 2–3 weeks for the remainder of treatment.^43^ Based on the sometimes profound blood pressure dynamics, however, more frequent blood pressure measurements (daily) might be required, and a baseline measurement is mandatory.

The same panel recommended less than 140/90 mmHg as the goal for blood pressure control in patients on VEGF inhibitor therapy in general and less than 130/80 mmHg for patients with diabetes and/or chronic kidney disease.^43^ This was in keeping with the Joint National Committee VII guidelines at the time, which since then have changed, (ie Joint National Committee VIII metrics: 150/90 mm Hg or higher in patients 60 years and older, or 140/90 mmHg or higher in patients younger than 60 years or with diabetes). Indeed, a more lenient approach with a goal of 150/100 in a phase II trial protocol was shown to be safe and effective.^44^ On the other hand, the systolic blood pressure intervention trial (SPRINT) trial data would suggest a benefit from a more aggressive approach with a systolic blood pressure goal of <120 mmHg (rather than the outline standard goal of <140 mmHg systolic).^45^ Even though on average, the intensive therapy cohort remain just around 120 mmHg, a 25% reduction in the primary endpoint of myocardial infarction (MI), acute coronary syndrome, stroke, heart failure, and death from CV causes emerged after 1 year. This was driven by a 38% reduction of heart failure events and a 43% reduction of CV death. Overall mortality was also reduced significantly by 27%. Patients without chronic kidney disease faced a 3.5 times higher risk of glomerular filtration rate decline by 30% – <60 mL/min/m^2^. Importantly, patients were required to be at increase CV risk by one or more of the following: clinical or subclinical CVD other than stroke; chronic kidney disease with an estimated glomerular filtration rate of 20 to less than 60 mL per minute per 1.73 m^2^ of body surface area, a 10-year risk of CVD of 15% or greater on the basis of the Framingham risk score; or an age of 75 years or older; patients with diabetes mellitus or prior stroke were excluded. Based on the results of this and several other trials, as well as meta-analyses on this subject, the American Heart Association along with 10 other societies released the 2017 guidelines for the prevention, detection, evaluation, and management of high blood pressure in adults.^46^ These guidelines recommend the use of BP-lowering medications for secondary prevention of CV events in patients with clinical CVD and an average systolic blood pressure of 130 mmHg or higher or an average diastolic blood pressure of 80 mmHg or higher, and for primary prevention in adults with an estimated 10-year atherosclerotic cardiovascular disease (ASCVD) risk of 10% or higher and an average systolic blood pressure 130 mmHg or higher or an average diastolic blood pressure 80 mmHg or higher.^46^

**Figure 4:**
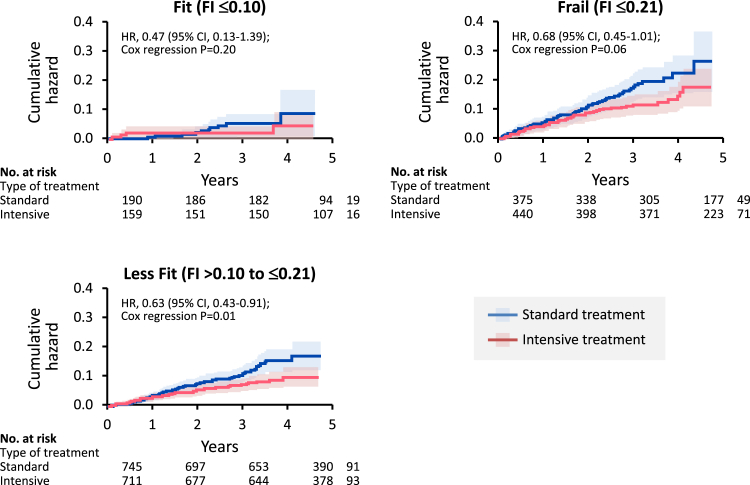
Kaplan–Meier curves for the primary CVD outcome in systolic blood pressure intervention trial (SPRINT) in participants aged 75 years or older by baseline frailty status.

We propose that for cancer patients on VEGF inhibitor therapy, the same strict blood pressure goals should be pursued. This is not limited to secondary prevention efforts, and we would argue that these patients meet primary prevention criteria and face a 10-year risk of CVD of 15% or more by SPRINT criteria. In a study of prostate cancer patients undergoing therapy with bevacizumab, a history of myocardial infarction, angina, peripheral arterial disease, arterial thrombosis, transient ischemic attack, or stroke increased the risk of an ATE by a factor of 2.29 (0.83–5.68). In comparison, bevacizumab treatment was a stronger and even more robust predictor (3 [1.25–7.19], *P* = .01).^47^ Furthermore, the event rate of ATEs was 3.8% over 21 months, which calculates into an event rate of 21.7% over 10 years. In metastatic cancer patients undergoing bevacizumab therapy, 5% developed an ATE over 15 months, which calculates into a 10-year event rate of 40%.^48^ Finally, the ATE event rate attributed to bevacizumab in the Japanese Adverse Drug Event database was 3.8% over 12 months, which translates into 38% in 10 years.^49^ For VSP-TKIs, a meta-analysis on sunitinib and sorafenib concluded on an ATE incidence event rate of 1.4% over 6.8 months, which equates a 10-year risk of 24.7%.^50^ Accordingly, on bevacizumab or VSP-TKI, cancer patients face a rate of ATEs that is well within the high risk range. What is unknown at this point is if strict blood pressure control in these patients will lead to fewer events and improved overall outcomes. The potential for adverse events needs to be acknowledged, and these patients will need close follow-up on blood pressure, renal function, and any evolving signs and symptoms of CVD. Importantly, the SPRINT trial does indicate that elderly and frail patients do not fare worse but better with stricter blood pressure control (Figure 4).^51^

(Reproduced from^51^).

**Figure 5:**
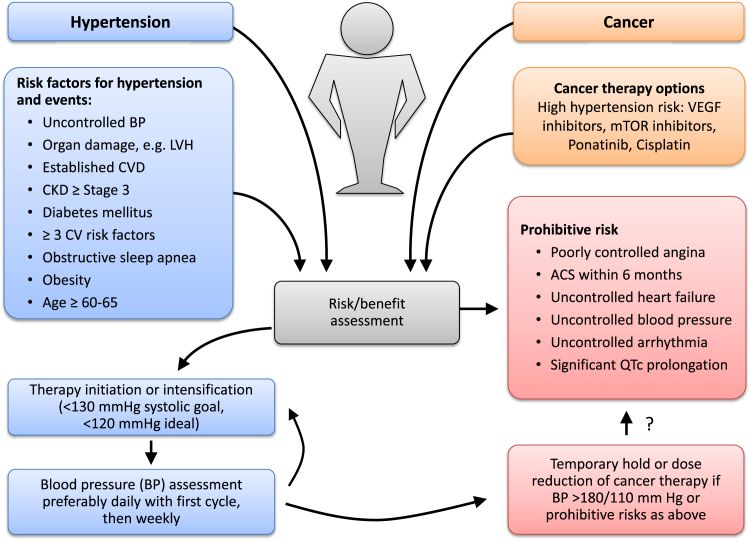
Evaluation proposal for cancer patients undergoing chemotherapy with hypertension risk such as those targeting the vascular endothelial growth factor (VEGF) pathway. Baseline evaluation should take into account risk factors for cardiovascular (CV) events, including uncontrolled blood pressure (BP), left ventricular hypertrophy (LVH), cardiovascular disease (CVD), chronic kidney disease (CKD), diabetes. Ideally patients should be optimized before starting chemotherapy and should be followed more closely early after starting therapy. In case of severe BP elevation or complications related or aggravated by it, cessation of therapy is to be considered. As outlined in the text, we propose a blood pressure goal for patients on VEGF inhibitor therapy of <130 mmHg systolic (2017 hypertension guideline) and <120 mmHg systolic ideally (SPRINT trial target).

Every patient, who is to start on VSP inhibitor therapy, should undergo a thorough evaluation of baseline condition and comorbidities (Figure 5). Ideally, blood pressure goals should be accomplished before the initiation of VEGF inhibitor therapy. In those on antihypertensive therapy, medication and diet adherence should be verified otherwise these are to be intensified. The risks of delaying anticancer therapy for optimization of the CV status always need to be balanced with the hazards of incomplete control or suboptimal management of CVD and risks. For blood pressure control, shorter acting agents with close follow-up (as frequently as every 2–4 days) might achieve goals quickly, and therapy can thereafter be transitioned to equivalent doses of longer acting agents.

The choice of the specific antihypertensive agent relates to the underlying mechanisms of systemic hypertension. Diuretics address the aspect of increased salt sensitivity, whereas NO donors address NO deficiency. Nitrates have been used successfully in these patients for resistant hypertension.^52^ Other vasodilators such as the calcium channel blocker nifedipine effectively reversed increases in blood pressure in VSP inhibitor-treated patients.^53^ Importantly, nifedipine did not negatively affect antitumor activity, which is of relevance as it may increase VEGF levels.^33^ The latter relates to the induction of CYP3A4, an effect even more so seen with the calcium channel blockers diltiazem and verapamil, which should be avoided in these patients. Amlodipine has thus been advocated as an alternative, which similarly prevented the early increase in blood pressure with VEGF inhibitor therapy.^54^ Consistent with the view of a mechanistic role of the ET-1 system in the hypertensive response to VEGF inhibitor therapy, dual ETa/ETb receptor blockade might be a valid yet not commonly taken approach. There is no strongly supported role for beta blocker as a class of drugs in these patients unless concomitant cardiac ischemia and evidence for adrenergic stimulation. There might, however, be a role for nebivolol as it increases endothelial NO production and may counteract the VEGF inhibitor effect. On the other hand, the same effect might be observed in the tumor vasculature. Carvedilol, based on its additional vasodilatory effect, beneficial action on endothelial cells, and reported antiangiogenic effects, seems to be the beta blocker of choice in this patient population.^55^, ^56^, ^57^

The role of angiotensin converting enzyme (ACE) inhibitors is somewhat a matter of debate. In agreement with a lesser role of the renin-angiotensin system in the mechanism of VSP inhibitor-induced hypertension, captopril was effective in lowering lower (10 mmHg) but not higher (35–50 mmHg) degrees of blood pressure elevations in animal models.^53^, ^54^ Comparable to dual ETa/ETb receptor blockade, captopril did prevent renal structural changes and is a preferred option in patients with proteinuria. Furthermore, four studies in 2014/2015 (three retrospective single center studies and one meta-analysis of 12 phase II and III clinical trials) indicated superior survival outcomes of patients with metastatic renal cell carcinoma, who were on angiotensin system inhibitors.^58^, ^59^, ^60^, ^61^ Furthermore, in cell culture experiments, captopril and lisinopril had an additive benefit to sunitinib in decreasing the viability of renal carcinoma cell lines. These results are in agreement with better outcomes of mice with renal cell carcinoma subjected to renin angiotensin system (RAS) inhibition.^58^, ^59^, ^60^, ^61^ However, not all studies are in agreement with a superior action of RAS inhibitors.^62^

**Figure 6:**
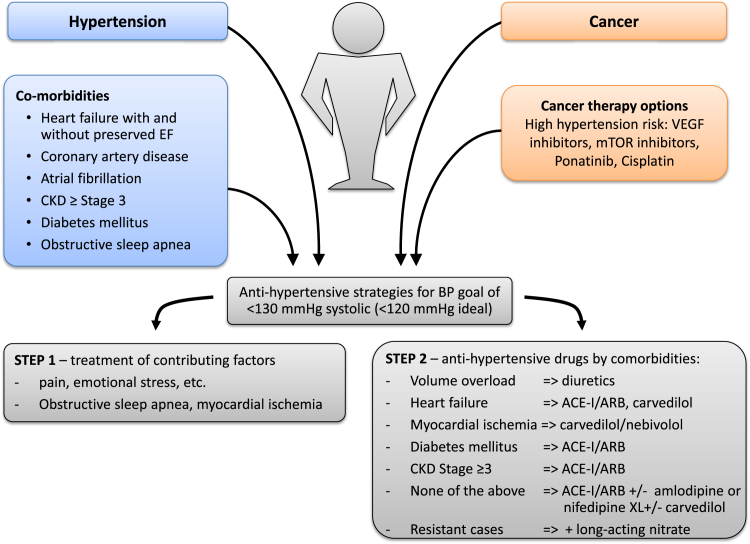
Management proposal for blood pressure (BP) control of cancer patients undergoing chemotherapy with hypertension risk. As outlined in the text, we propose that patients on VEGF inhibitor therapy should be treated toward a goal of <130 mmHg systolic (2017 hypertension guideline) and <120 mmHg systolic ideally (SPRINT trial target). Two steps toward reaching this goal are to be pursued: (1) treatment of contributing and aggravating factors and (2) antihypertensive therapy by comorbidity.

In keeping with the outlined data and the overall goal to improve endothelial function and NO availability and to decrease pathological angiogenesis, one may argue that ACE inhibitors ± amlodipine is the preferred first line therapy for patients with new onset or worsening hypertension on VSP inhibitor therapy in the absence of any other comorbidity that would direct therapy. Carvedilol may be considered as a third step based on the additional beneficial vascular actions. For blood pressure control in patients with refractory hypertension, diuretics +/- nitrates may enhance the vasodilatory approach. An outline of a potential approach to therapy is provided in Figure 6.

## Clinical Burden of Altered Vasoreactivity, Atherosclerosis, and Arterial Events with VSP Inhibitors

While hypertension is the most notable vascular effect, patients on VSP inhibitor therapy also encounter a higher risk of ischemic and thrombotic events. Indeed, chest pain episodes have been reported in 1%–15% of patients, ranging from stable angina to acute coronary syndrome.^16^ Meta-analyses confirm a 1.5 and 2–3 times higher risk of ATEs with bevacizumab and VEGF receptor TKIs, respectively.^13^ In one of the first of these analysis of four phase II and one phase III trial in patients with colorectal, breast, and non-small cell lung cancer, the overall incidence of ATEs was 3.8% versus 1.7% in bevacizumab-treated versus non-treated patients (incidence of fatal ATEs within 30 days 0.6% vs. 0.3%), calculating into a relative ATE risk of 2 (95% confidence interval [CI] 1.1–3.8).^48^ The ATEs accounted for included angina, arterial thrombosis, myocardial infarction, myocardial ischemia, cerebrovascular accident, cerebral infarct, and cerebral ischemia. In time-dependent analyses, the events seem to continue to accumulate over time but with a 5% event rate in the first year and a 2.5% event rate in the second year. In agreement with an earlier rather than later onset, the median time to event was only 2–3 months. Myocardial (angina and myocardial infarction) and cerebrovascular (transient ischemic attack and stroke) events accounted for 40% of the total number of events each. Bevacizumab was confirmed as an independent risk factor (hazard ratio [HR] 1.95, 95% CI 1.04–3.67) as was age ≥ 65 years (HR 2.2, 95% CI 1.2–4). A history of ATE (including angina pectoris, arterial thrombosis, cerebral infarct, cerebral ischemia, cerebrovascular accident, myocardial infarction, and myocardial ischemia) was the strongest independent risk factor for an ATE on VSP inhibitor treatment (HR 3.65, 95% CI 1.9–6.9). These risks may be additive, as patients with the combination of all three had an 8.3-fold increased risk of ischemic events. In the univariate analysis, for those with a history of ATE, a history of MI carried the strongest risk (HR 4.9, 95% CI 2.6–9.4). Hypertension at baseline was a weaker risk factor (HR 1.89, 95% CI 1.1–3.3), and neither MI nor hypertension was an independent predictor of ATEs. Of further interest, patients with a history of ATE had the poorest overall and progression-free survival.^48^

Two subsequent meta-analyses on 20 RCTs in patients with colorectal, breast, non-small cell lung, renal cell, and pancreatic cancer (all but one being the same) reached the same conclusions.^63^, ^64^ All grade ATEs occurred in 3.3% of the patients on bevacizumab, grade 3 or higher in 2%, and fatal in 0.4% (five strokes, two MIs). The calculated relative risks (RRs) for all grade ATEs were 1.44 in one and 1.46 in the other study (95% CI 1.1–1.9). The highest incidences were noted in colorectal cancer patients (3%–6%), which constituted the largest group of patients. Among the different types of ATEs, the highest risk was noted for high-grade cardiac ischemia (summary incidence 1.5%, RR 2.1 [95% CI, 1.12–4.08]). There was no difference in risk by treatment duration or dose intensity.

A subsequent meta-analysis of seven phase II and III trials RCTs, looking specifically at myocardial ischemic endpoints (myocardial infarction, unstable angina, coronary revascularization, coronary artery disease (CAD), arrhythmias, sudden cardiac death, or CV-related death) and including two more studies than in the analysis by Ranpura et al., confirmed an overall RR of 2.5 (95% CI 1.4–4.5). The total event rate was low, only 1%, and differed between low versus high-dose bevacizumab therapy (RR 2.1 [95% CI 1.1–4.2] vs. 4.8 [95% CI 1.0–22.4]). An elevated risk was most consistently noted in colorectal cancer patients (RR 2.1 [95% CI 1.1–4.1]).^65^

Cerebrovascular events have also been demonstrated in cancer patients on VSP inhibitor therapy. In a meta-analysis of 16 RCTs on cerebrovascular events in phase II and III trials with bevacizumab, the overall incidence was relatively low at 0.5%, but the RR was 3.3 (95% CI 2–5.5), again highest in metastatic colorectal cancer patients (RR 6.4 [95% CI 1.2–35.6]).^66^ This analysis was able to further stratify by stroke type, based on 11 RCTs on ischemic stroke and seven RCTs on hemorrhagic strokes. The ischemic stroke incidence was 0.5% (RR 3.2 [95% CI 1.7–6.1]), highest in metastatic mesothelioma (1.9%). The hemorrhage stroke incidence was 0.3% (RR 3.1 [95% CI 1.4–7.0]), highest in glioblastoma (1.4%). A dose-dependent association was noted in this analysis as well (RR 1.96 [95% CI 0.8–5.1] vs. 3.97 [95% CI 2.2–7.4]) in low versus high-dose groups.^66^

With regard to VSP-TKIs, the first meta-analysis was conducted on 10 RCTs involving sunitinib and sorafenib with arterial thrombosis, cerebral infarct, cerebral ischemia, cerebrovascular accident, myocardial infarction, and myocardial ischemia as ATEs. The incidence was 1.4% overall and not significantly different between the two chemotherapeutics (RR 3 [95% CI 1.3–7.4]).^50^ A subsequent, more comprehensive meta-analysis in terms of trials (19 RCTs) and endpoints (arterial thrombosis/thromboembolism, cerebral infarct, cerebral ischemia, intestinal ischemia, ischemic stroke, CV accident, central nervous system (CNS) ischemia, intracardiac thrombosis, myocardial infarction, myocardia ischemia, defined by the NCI-CTC criteria) confirmed an overall ATE incidence of 1.5%.^67^ The highest ATE incidences were noted for cediranib (3.2%) and pazopanib (2.4%), and RRs were confirmed to be significantly elevated for pazopanib (4.6 [95% CI 1.1–18.7]) and sorafenib (2.3 [95% CI 1.2–4.4]) only. The largest number of events were again noted for cardiac ischemia/infarction in both TKI and control arm, and the RR was significantly elevated only for this endpoint (2.6, 95% CI 1.3–5.2). Only patients with renal cell carcinoma experienced a significantly elevated RR (3, 95% CI 1.4–6.5).^67^

## Mechanisms of Altered Vasoreactivity, Atherosclerosis, and Arterial Events with VSP Inhibitors

As outlined above, several studies have evaluated the impact of VSP inhibition on vascular reactivity, and the synthesis of these studies is that VEGF inhibitors induce an imbalance in endothelium-derived vasodilatory and vasoconstricting factors. Endothelial dysfunction is a term often used to describe this condition, and paradoxical vasoconstriction is one of its hallmarks.^68^ Furthermore, endothelial dysfunction has a facilitating action on coronary vasospasm, which, by strict definition, is a functional aberration on vascular smooth muscle cell level.^69^ Profound vasospasm has been confirmed in response to ergonovine and has been implicated in at least one reported case of sorafenib-induced myocardial infarction.^70^ Whether this relates to alterations in the activity of the Rho/Rho-associated protein kinase pathway or other kinases is not known at this point.^69^ Potential reasons that this has not been more commonly reported may include lack of testing and significant under recognition as well as inter-individual differences and predispositions, genetic and acquired.

**Supplementary Figure 2:**
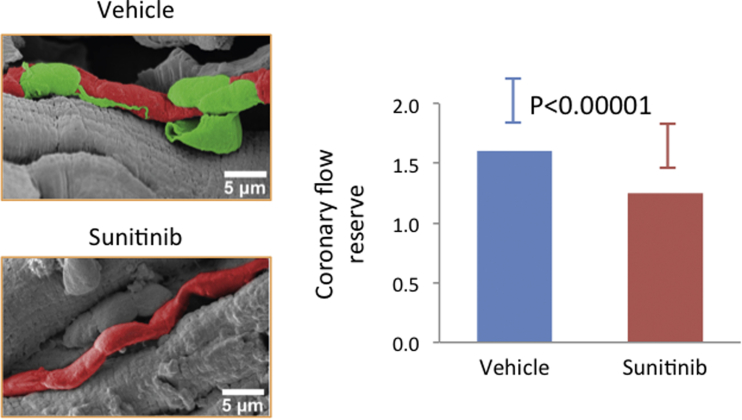
Illustration of the elimination of pericytes in the myocardial microcirculation and reduction of coronary flow reserve by sunitinib (Used with permission from Chintalgattu V, Rees ML, Culver JC, Goel A, Jiffar T, Zhang J, Dunner K, Jr., Pati S, Bankson JA, Pasqualini R, Arap W, Bryan NS, Taegtmeyer H, Langley RR, Yao H, Kupferman ME, Entman ML, Dickinson ME and Khakoo AY. Coronary microvascular pericytes are the cellular target of sunitinib malate-induced cardiotoxicity. *Sci Transl Med*. 2013;5:187ra69.).

Importantly, the vascular alterations seen with VSP inhibitor therapy extend into the microvasculature. Coronary microvascular dysfunction, that is, impairment of the endothelium-dependent and endothelium-independent vasodilatory response of the coronary microcirculation is noted after 8 days of sunitinib administration.^39^, ^40^ In addition, depletion of microvascular pericytes has been documented with sunitinib (Supplemental Figure 2). This may be due to the additional inhibition of PDGF signaling and adds another layer to endothelial and microvascular dysfunction.^71^ Not surprisingly, 70% of patients on sunitinib treatment have a reduced coronary flow reserve (on average 1.8 ± 0.4), especially with longer duration of therapy.^72^ The reduced coronary flow reserve can contribute to presentations of stable and unstable angina as well as to a reduced myocardial reserve. Acute functional alterations of the coronary microvascular have also been implicated in Takotsubo's. The microvasculature might be more susceptible to the effect of VEGF inhibitor therapy as under basal conditions human microvascular but not macrovascular endothelial cells constitutively generate and release VEGF.^73^

**Supplementary Figure 3:**
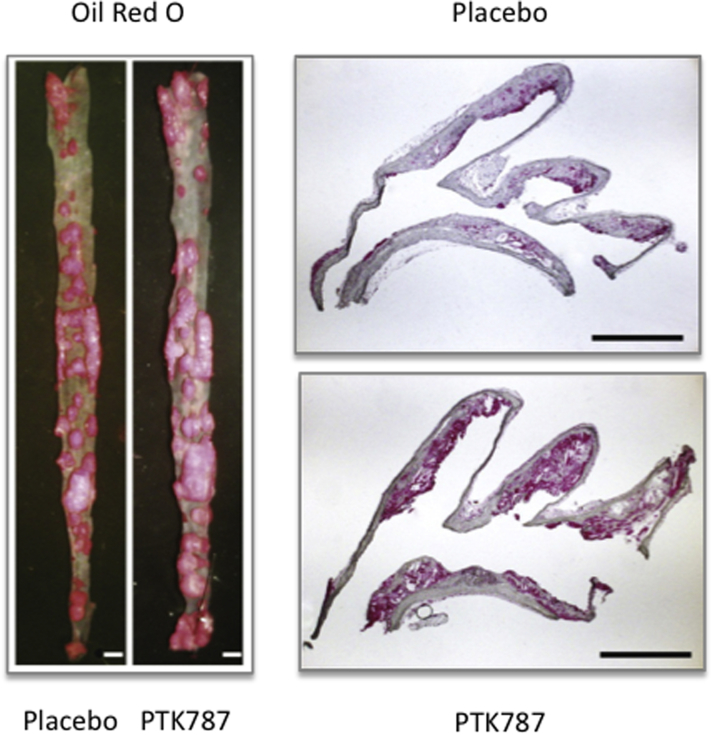
Examplatory display of the pro-atherosclerotic effects of the use of a pan-VEGF inhibitor (PTK 787) in Apo E-deficient mice. En face aortas (left panel) show marked increase in stained lipid areas. Cross-sections on the level of the aortic arch stained with Oil Red O furthermore highlight the differences in atherosclerotic plaque burden. (Used with permission from Winnik S, Lohmann C, Siciliani G, von Lukowicz T, Kuschnerus K, Kraenkel N, Brokopp CE, Enseleit F, Michels S, Ruschitzka F, Luscher TF and Matter CM. Systemic VEGF inhibition accelerates experimental atherosclerosis and disrupts endothelial homeostasis--implications for cardiovascular safety. *Int J Cardiol*. 2013;168:2453-61.).

This being said, arterial endothelial cell culture experiments showed that exposure to a pan-VEGF receptor TKI increases mitochondrial superoxide generation with uncoupling of endothelial nitric oxide synthase (eNOS) and subsequent imbalance in endothelial superoxide and NO production.^74^ This profound alteration in endothelial function predisposes to atherosclerosis, and indeed, cases of progression of CAD and plaque rupture have been reported for sorafenib and sunitinib, respectively.^75^, ^76^ In in vivo experiments, pan-VEGF inhibition markedly accelerated preexisting atherosclerosis yet without an increase in features of plaque vulnerability (Supplemental Figure 3).^74^ These findings are truly intriguing as the effects of VEGF and VEGF inhibitors on atherosclerosis have not been without debate. For instance, another experimental study found that bevacizumab reduced plaque neovascularization and growth similar to the initial studies with angiogenesis inhibitors.^16^ The vascular effects of VEGF and VEGF inhibition may depend on local VEGF concentrations more than recognized, with low levels necessary for vascular homeostasis and high concentrations resulting in vasculoproliferative effects. Such dose dependency may account for the opposing effects described in different studies and settings. Collectively, one may conclude though that inhibition of physiological concentrations of VEGF is associated with accelerated progression of atherosclerosis, whereas inhibition of supra-physiological levels is of therapeutic value.

Endothelial dysfunction also predisposes to perturbed hemostasis and vascular thrombosis.^68^ Hemostasis is normally regulated by a tight balance between pro-coagulant and anti-coagulant factors, pro-fibrinolytic and anti-fibrinolytic factors, and platelet-activating and platelet-inhibiting factors. The reduction in the production of NO and prostacyclins secondary to interference with the VSP translates into interference with antiplatelet effects of the endothelium.^77^ In an elegant study, cancer patients who developed thromboembolic events with VSP-TKI therapy were found to have a higher endogenous thrombin potential within 4 weeks of therapy and much higher circulating levels of soluble tissue factor and E-selectin at baseline with further increase- on therapy, which may henceforth serve as potential biomarkers of CV event risk in these patients.^78^ Tumors themselves secrete pro-inflammatory and pro-coagulant factors that can further promote thromboembolism. Increased blood viscosity is another mechanism that may contribute to thromboembolism when VEGF signaling is inhibited. Finally, VEGF inhibitors such as bevacizumab may act in a mechanism similar to heparin-induced thrombocytopenia. VEGF, like PF4, binds heparin and in immune complexes with bevacizumab can bind to FCyRIIa inducing aggregation and pro-coagulant activity.^79^

## Monitoring and Management of Altered Vasoreactivity, Atherosclerosis, and Arterial Events with VSP Inhibitors

Based on the framework provided in their hypertension assessment and management document, the Cardiovascular Toxicities Panel of the National Cancer Institute extended the recommendations to address the aspects outlined above.^80^ However, these essentially focus on follow-up electrocardiograms (ECGs) once on therapy, not only to monitor for QTc prolongation but also for ischemic ECG changes. The specific recommendations are for an ECG at baseline, then at 2–4 weeks, 8–12 weeks, and finally every 3 months. If ischemic ST-T wave changes were to be noted, a cardiac evaluation is advised and discretion should be used in regard to continuation of therapy. If the patient develops angina (or an abnormal stress test or angiogram) or suffers an acute myocardial infarction, VEGF inhibitor therapy is to be discontinued. It was further recommended not to start VEGF inhibitor therapy in patients with unstable or poorly controlled angina/myocardial ischemia at baseline or a recent myocardial or arterial thrombotic event (and neither in those with uncontrolled heart failure, uncontrolled arrhythmia, significant QTc prolongation, and uncontrolled hypertension). Recommendations for a more definitive work-up of (clinically silent and yet significant) ASCVD in patients considered for VEGF inhibitor therapy were not provided. Neither was specific guidance suggested on how to manage these patients through their therapy.

The merit of vasoreactivity testing, ankle-brachial-index, or carotid-intima media thickness for risk stratification and the prediction of ATEs with VSP inhibitor therapy are unknown. Likewise, the predictive value of baseline and follow-up coronary computed tomography angiography and noninvasive stress testing is not defined in this population, neither for gauging the progression of CAD nor for predicting acute arterial thrombotic events. Another question is if those on VSP inhibitor therapy should be considered as high risk, as discussed above. All of these questions are pertinent only as much as the cancer therapy allows these patients to outlive and thus to experience the time dynamics of these adverse vascular events, most concerningly acute myocardial infarction and stroke.

**Supplementary Figure 4:**
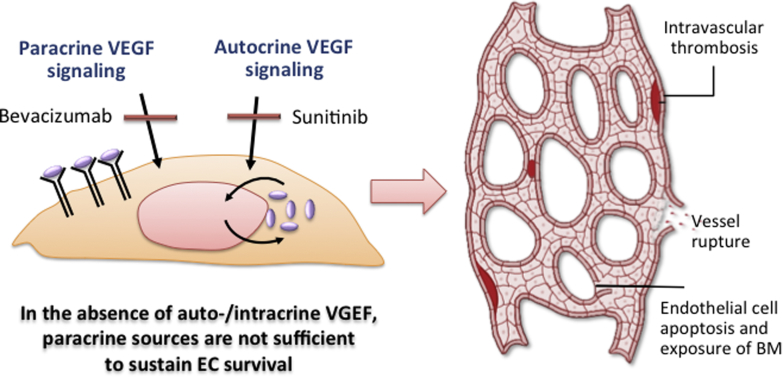
Illustration of the principle distinction between paracrine and autocrine VEGF signaling and the effects of its inhibition on endothelial function and survival. Downstream consequences range from thrombotic to hemorrhagic events, the former being more common.(Used with permission from Lee S, Chen TT, Barber CL, Jordan MC, Murdock J, Desai S, Ferrara N, Nagy A, Roos KP and Iruela-Arispe ML. Autocrine VEGF signaling is required for vascular homeostasis. *Cell*. 2007;130:691-703.).

Retrospective analyses have outlined that the incidence of ATEs on bevacizumab therapy is significantly attenuated by aspirin in the high-risk group of patients ≥65 years with a prior ATE history (12.5% vs. 22.9% in aspirin nonusers).^48^ This comes at the cost of a difference in grade 3 and 4 bleeding events of 1.1% versus 0.5% in patients receiving and not receiving bevacizumab, respectively.^48^ The exact mechanisms of hemorrhage with VSP inhibitor therapy are unclear but may relate to more profound endothelial damage and endothelial cell apoptosis as well as platelet dysfunction and are most frequently seen in patients with gastrointestinal tract or lung cancers.^81^ In elegant experimental studies, it was furthermore shown that various stressors such as oxidative stress and hypoxia trigger the activation of the VSP by both autocrine and intracrine VEGF sources. The later is critical for endothelial survival and if impaired by VSP-TKIs, endothelial cell apoptosis may evolve with the consequences depending on the environment, that is, hemorrhage and thrombosis in smaller vessels without and larger vessels with an underlying basement membrane (Supplemental Figure 4).^82^

Taking the bleeding risk into consideration, it is unlikely that patients are advised to take dual antiplatelet therapy unless demanded by prior drug-eluting stent placement. In addition to medications outlined for optimal blood pressure control, the most potent intervention for vascular health and prevention in these patients may be statin therapy. Statins have been shown to improve NO bioavailability and eNOS activity. They have been shown to activate phosphatidylinositol-3 kinase and AKT signaling, which is suppressed by VEGF. Thus, there have been concerns that statins may reduce the antiangiogenic and anti-tumor efficacy of VEGF inhibitor. However, statins exert concentration- and cell type-dependent effects that need to be taken into consideration. Statins reduce the constitutive VEGF synthesis of microvascular endothelial cells, whereas they upregulate VEGF expression in macrovascular endothelial cells. Furthermore, at low concentrations, they are pro-angiogenic, whereas at high concentrations, they are anti-angiogenic.^73^, ^83^ These dual dynamics have been recapitulated in tumor models, adding to the increasing view of anticancer effects of statins.^84^, ^85^ Accordingly, there should be a low threshold for the use of high-dose statin therapy in cancer patients who are to or are already undergoing VEGF inhibitor therapy and are at CV risk.

The benefit may be enhanced by the use of amlodipine, which is often required for blood pressure control in these patients as outlined above anyway and has been shown to reduce CV events in patients with mild CAD and even normal blood pressure along with preventive effects on coronary artery plaque progression.^86^ Calcium channel blockers and statins may act synergistically in halting the progression of atherosclerosis as shown in the Regression Growth Evaluation Statin Study as well as in experimental models.^87^ The combination of atorvastatin and amlodipine has been shown to improve acute NO release and endothelial function as well as arterial compliance in hypertensive hyperlipidaemic patients, which seems to be what is needed in CV risk patients on VSP inhibitor therapy.^88^

Finally, and even though difficult to accomplish in a cancer population, exercise should be advocated to promote endothelial and vascular health. This is mediated by increase in shear stress, a potent natural inducer of eNOS via the protein kinase B/AKT pathway. Shear stress-related effects during exercise are of relatively greater impact for the macrovasculature (and should thus spare the tumor microvasculature), even though restores the endothelial response to VEGF and improves endothelium-dependent vasodilatation in epicardial coronary vessels and in resistance vessels.^89^, ^90^, ^91^ Exercise has the additional advantage of improving the CV reserve in general as well as alerting to any of its potential impairments much sooner than detected by resting assessments. Accordingly, for patients with high-CV risk, especially those with atherosclerotic disease and particularly anyone with a history of clinically evident CAD, CVD, and peripheral arterial disease should be on a baby aspirin, high-dose statin, possibly in combination with ACE inhibitor and/or amlodipine therapy and should be asked to exercise routinely (goal for aerobic exercise is 3–5 d/wk, 150+ min/wk of moderate intensity).

If and when to resume cancer therapy in patients who have experienced a cardiac event is not defined. Clinical trials excluded patients with such an event within 6–12 months of initiation of treatment and often with any clinically significant CVD.^43^ Angiogenesis is part of the natural healing response after an acute ischemic event such as myocardial infarction, and vascular proliferation reaches a plateau after 2 weeks at which point in time the risk of hemorrhages declines.^92^ One may wish to wait until the response to injury is relatively complete, that is, at least 6 weeks and preferably 90 days. Impaired angiogenesis also contributes to limb ischemia and reduced wound healing, for example, after coronary or peripheral artery bypass grafting. Mechanistically one may further argue that the effects of VEGF inhibition might be most detrimental in those patients in whom the system is up-regulated. This would include patients at risk or in a state of general ischemia (eg obstructive sleep apnea, reduced cardiac output, or severe anemia) or with more localized tissue ischemia (eg severe or unstable angina or transient ischemic attack).

## Summary

Antiangiogenic therapy such as VSP inhibition has improved outcomes for several cancers but not without adverse effects. These include vascular toxicities, which were to be expected based on the cardinal role of VEGF in vascular function and health. At present, however, there are no studies to support any specific strategy, and expert consensus recommendations are nearly 10 years old. Hypertension is the most commonly seen “vascular side effect” in patients undergoing VSP inhibitor therapy, but again, the best management approach to these patients is not defined. The SPRINT trial, conducted in a patient population at higher CV risk, advocates for more intensive blood pressure targets (<120 mmHg systolic), and one may argue that patients undergoing VSP inhibitor therapy are by default at a higher CV risk based on VEGF-related impairment in endothelial function and should be more intensively treated for this reason. Endothelial dysfunction promotes vasoconstriction, atherosclerosis, platelet activation, and coagulation, all in favor of ATEs observed in this patient population. Patients with a prior ATE and ≥65 years of age are seemingly at highest risk. They in particular, but preferably anyone with a increased CV risk as defined, for instance, by the ASCVD risk score or prior CV history should receive optimal medical therapy with aspirin, ACE inhibitor, amlodipine, high-dose statin therapy (Graphical Abstract). Exercise is furthermore promoted to improve CV health in these patients. The controversial nature of a more intensive management proposal is understood, but these patients now live long enough that CV complications can have a significant impact and so can preventive measures.

## Figures and Tables

**Supplementary Figure 1:**
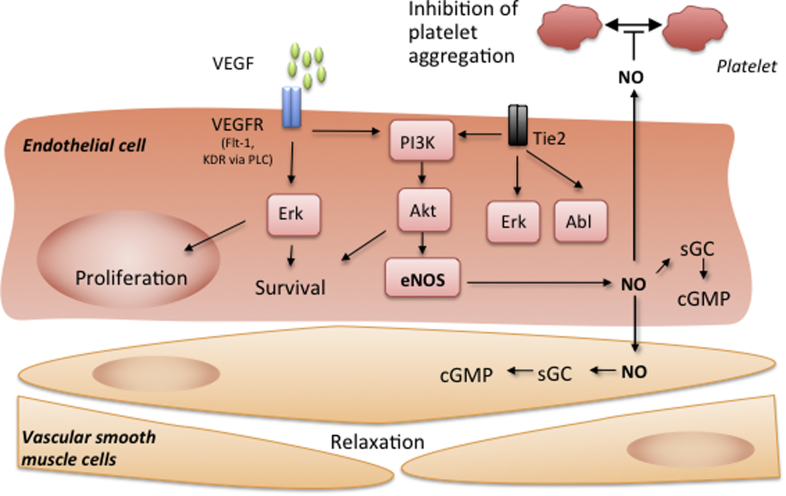
Outline of the pathways stimulated by VEGF in endothelial cells and downstream consequences.
